# INCIDENCE OF URINARY TRACT INFECTION AFTER CYSTOGRAPHY

**DOI:** 10.1590/1984-0462/2021/39/2019386

**Published:** 2020-11-20

**Authors:** Joana Sousa Martins, Margarida Pinto, Manuela Braga, Paulo Calhau

**Affiliations:** a Pediatric Service at Hospital Garcia de Orta, E.P.E., Almada, Portugal.

**Keywords:** Cystography, Pyelonephritis, Vesico-ureteral reflux, Pediatrics, Cistografia, Pielonefrite, Refluxo vesicoureteral, Pediatria

## Abstract

**Objective:**

Cystography an invasive procedure with potential complications such as urinary infection (UI). There are few studies about the incidence of complications associated with this procedure. The purpose of this study is to evaluate the incidence of post-cystography urinary infection (UI.).

**Methods:**

Retrospective study with a review of clinical records of patients under 15 years of age, followed in this hospital, who underwent cystography (radiologic or indirect radionuclide) between 2009 and 2018. Post-cystography UI was defined when it occurred until seven days after the procedure. Descriptive and nonparametric statistics were applied to assess possible predictive factors related with post-cystography UI.

**Results:**

In the study period, 531 cystograms were undertaken (55% indirect radionuclide and 45% radiologic). The mean age at the procedure was 11.5 months; 62% were boys. Every patient had a previous negative urine culture; 50% were under antibiotic prophylaxis at the time of the procedure. The most common indication for the procedure was the post-natal study of congenital hydronephrosis/other nephrological malformation (53%), followed by the study of febrile UI (31%). Vesicoureteral reflux (VUR) was diagnosed in 40% of procedures. Post-cystography UI occurred in 23 cases (incidence of 4.3%). The most frequent microorganism was *E. coli* (52%). The presence of VUR was significantly associated with the occurrence of post-cystography IU.

**Conclusions:**

The incidence of post-cystography UI was low in our sample. The presence of VUR was significantly associated with the occurrence of post-cystography UI. The authors highlight the importance of an adequate catheterization technique and the need for clinical surveillance after the procedure.

## RESUMO

**Objetivo:**

A cistografia é um exame invasivo que apresenta potencial iatrogenia, nomeadamente infecção urinária (IU). Os estudos sobre a incidência de complicações associadas a esse exame são escassos. O objetivo deste trabalho foi avaliar a incidência de IU após realização de cistografia.

**Métodos:**

Estudo retrospetivo por consulta dos prontuários clínicos dos doentes com idade inferior a 15 anos, seguidos em consulta nesse hospital, que realizaram cistografia (radiológica ou isotópica) entre 2009 e 2018. Admitiu-se relação de causalidade quando o diagnóstico de IU ocorreu até sete dias após a realização do exame. Foi realizada análise estatística descritiva e utilizados testes não paramétricos para avaliar possíveis fatores preditores da ocorrência de IU após cistografia.

**Resultados:**

Realizaram-se 531 cistografias (55% isotópicas e 45% radiológicas). A mediana de idade foi de 11,5 meses; 62% eram do sexo masculino. Todos os doentes efetuaram urocultura prévia (negativa); 50% recebiam profilaxia antibiótica (ATB) à data do exame. A indicação mais frequente foi o estudo pós-natal de hidronefrose (HN) congênita/outra malformação nefrourológica (53%), seguida do estudo da IU febril (31%). Documentou-se refluxo vesicoureteral (RVU) em 40% dos exames. Ocorreu IU após cistografia em 23 casos (incidência de 4,3%). O microrganismo mais frequente foi a *E. coli* (52%). Verificou-se associação entre a presença de RVU e a ocorrência de IU.

**Conclusões:**

A incidência de IU pós-cistografia foi relativamente baixa na amostra deste estudo. Observou-se associação entre a ocorrência de IU após cistografia e a presença de RVU. Sublinha-se a importância de uma técnica adequada de cateterização vesical e da vigilância clínica após o exame.

## INTRODUCTION

Cystography is an ancillary diagnostic test often used in Pediatrics, being mostly recommended to diagnose vesicoureteral reflux (VUR). In the past, the imaging investigation after the occurrence of urinary infection was the most common indication for its performance among infants and children; nowadays, it is known that 25 to 30% of the children who present a first episode of febrile urinary infection (UI) have VUR.^1^ With the advance in prenatal echographic diagnosis, cystography is more frequently carried out in the post-natal evaluation of asymptomatic children with prenatal diagnosis of moderate or severe hydronephrosis (HN) - and VUR is detected in up to 15% of these cases - or other renal-urological disorders.^2^ ^,^ ^3^

Cystography is an invasive procedure that includes vesical catheterization and presents potential iatrogenic complications, such as UI. The existing studies on the incidence of UI associated with this test are scares and have very different methodologies, since incidence ranges between 0 and 30%.^2^ ^,^ ^4^ ^,^ ^5^ ^,^ ^6^ ^,^ ^7^ ^,^ ^8^ ^,^ ^9^ ^,^ ^10^ The objective of this study was to assess the incidence of post-cystography UI in the ediatric population.

## METHOD

Retrospective study conducted after reviewing clinical charts of 386 patients who had undergone radiology or isotope cystography in Hospital Garcia de Orta (tertiary hospital in the city of Almada, Portugal), between January 1^st^, 2009, and December 31^st^, 2018. We only considered patients aged less than 15 years (since until 2015 the hospital only received patients up to this age). Patients who were not followed-up in this institution were excluded.

Clinical and demographic data of the analyzed children were obtained, as follows: age, sex, indication for a cystography, specialty that requested this test, previous history of UI, antibiotic prophylaxis at the date of the examination, result of the cystography (presence of absence of VUR), and occurrence of UI after the test. The causality relationship was admitted when the UI was diagnosed up to seven days after the examination. Routine urine analysis and posterior urine culture were performed for all patients who, in the referred period, presented with symptoms that suggested UI (fever, irritability, vomit, dysuria, pollakiuria). UI was defined as the presence of suggestive clinical symptoms, characteristic routine urine analysis and positive urine culture (the following bacterial counts were considered: if suprapubic aspiration, any growth; if bladder catheterization, presence of ≥5^4^ of colony forming units/mL; if mid-stream urine collection , presence of ≥10^5^ of colony forming units /mL). In 100% of the children, urine culture was carried out up to 36 hours before the examination, which was negative in all cases.

Statistical analysis was performed using the software SPSS® Statistics 24 (IBM Corp., 2016, the United States). Descriptive analysis was used to characterize the sample and nonparametric tests to assess predictive factors of the occurrence of post-cystography UI. A 0.05 statistical significance level was considered. The study was approved by the Ethics Commission of Hospital Garcia de Orta.

## RESULTS

During the study period, there were 531 cystograms, being 293 isotope (55%) and 238, radiology (45%). The mean age of the patients was 11.5 months. Sixty-two percent of the exams were performed in male children. The clinical characteristics regarding the conditions of the cystography are described in Table 1. The most frequent indication for the cystography was the post-natal analysis of moderate or severe congenital HN (with or without associated ureteral dilatation) or another renal-urological malformation (n=282, 53%), followed by the study of febrile UI (n=176, 33%), according to the protocol of our institution (criteria to perform cystography after febrile UI: echography with moderate to severe pyelocaliceal dilatation, diagnostic imaging with function differential >20%, recurring febrile UI, micro-organism that is not *E.coli*). At the date of the examination, 268 patients (50%) had previous prescription of antibiotic chemoprophylaxis. Most cystograms (n=400, 75%) was requested in a Pediatric Nephrology appointment.

**Table 1:** Indication of cystography and use of antibiotic prophylaxis.

	n (%)
Indication for the exam (n=531)	
PND of congenital HN or another renal-urological disorder	282 (53.1)
Work-up for febrile UI	176 (33.2)
Spina bifida	33 (6.2)
HN or another renal-urological disorder without PND	15 (2.8)
Others*	25 (4.7)
Antibiotic prophylaxis at the date of the exam (n=268)	
Isolated trimetoprim	256 (95.5)
Nitrofurantoin	7 (2.6)
Amoxicillin+clavulanic acid	3 (1.2)
Co-trimoxazole	2 (0.7)
Specialty in charge of requesting the exam (n=531)	
Pediatric Nephrology	400 (75.3)
General Pediatrics	103 (19.4)
Pediatric Surgery	25 (4.7)
Others	3 (0.6)

PND: pre-natal diagnosis; HN: hydronephrosis; UI: urinary infection; VUR: vesicoureteral reflux; *Others: hyspopadias, n=11; sibling diagnosed with VUR, n=4; enuresis, n=4; *pollakiuria*, n=1; traumatic spinal cord injury, n=1; sexual ambiguity, n=1; hematuria, n=1; urachal cyst, n=1; acute encephalomyelitis - urinary retention, n=1.

The presence of VUR was documented in 215 exams (40%), which corresponded to 311 reflux units, being 52% left, 42%, level I or II; 35%, level III; and 23%, level IV or V (Table 2). Post-cystography UI occurred in 23 cases, which corresponds to a 4.3% incidence. The most frequent symptomatology was fever (70% of the cases) and lower urinary tract symptoms (22% of the cases). The most frequently isolated microorganism was *E. coli*, in 12 cases (52%). In two cases, in which *E. coli* was isolated, it was not possible to access the records about the presented symptomatology or the method for urine collection. In one of the cases (1-month-old infant with fever and routine urine analysis, obtained by suprapubic aspiration, with leukocyturia and nitrituria), it was not possible to obtain information about the isolated microorganism. The clinical characteristics of the patients and the examinations which showed post-cystography UI are listed in Table 3.

**Table 2:** Results of the cystograms with evidence of vesicoureteral reflux.

	n (%)
Unilateral/Bilateral VUR (n=215)	119 (55)/96 (45)
Laterality (n=311)	
Left	161 (52)
Right	150 (48)
Degree of reflux (n=311)	
I	13 (4)
II	117 (38)
III	108 (35)
IV	54 (17)
V	19 (6)

VUR: vesicoureteral reflux.

**Table 3:** Characteristics of the cystograms in cases with urinary infection after the procedure.

Characteristics	n (%) or median (variation)
Age at the exam (n=23)	4.5 months (0 months-12 years)
Sex (n=23)	
Male	16 (70)
Female	7 (30)
Type of cystography (n=23)	
Isotopic	12 (52)
Radiological	11 (48)
Presence of VUR (n=23)	14 (61)
Antibiotic prophylaxis at the exam (n=23)	13 (57)
Indication for the exam (n=23)	
Study of febrile UI	12 (52)
PND of congenital HN or other renal-urological disorder	9 (39)
Study of Spina bifida	1 (4,5)
Others (hypospadias)	1 (4,5)
Time (days) until UI (n=23)	3 dias (1-7 dias)
Symptomatology (n=21)	
Fever	16 (70)
Symptoms of the lower urinary tract	5 (22)
Vomit	4 (17)
Irritability	2 (9)
Type of urine collection (n=23)	
Suprapubic aspiration	11 (48)
Collector bag	4 (17)
Bladder catheter	2 (9)
Mid-stream urine	4 (17)
Unknown	2 (9)
Routine urine analysis (n=21)	
Leukocyturia	19 (90)
Nitrituria	10 (48)
Isolated microorganisms (n=23)	
*Escherichia coli*	12 (52)
*Proteus mirabilis*	3 (13)
*Klebsiella pneumoniae*	2 (9)
*Enterococcus faecalis*	2 (9)
*Enterobacter cloacae*	1 (4)
*Estafilococus lugdunensis*	1 (4)
*Pseudomonas aeruginosa*	1 (4)
Unknown	1 (4)
Need for hospitalization (n=23)	8 (35)
Days of hospitalization	3 days (1 to 11 days)

VUR: vesicoureteral reflux; UI: urinary infection; PND: pre-natal diagnosis; HN: hydronephrosis.

The relation between demographic and clinical characteristics and those related with cystography and the occurrence of post-cystography UI are detailed in Table 4. A higher percentage of cases with post-cystography UI presented evidence of VUR in the exam (61%), when compared to cases without UI (40%), and the difference was statistically significant (p=0.05). Of the cases with VUR, there was an association between the occurrence of post-cystography UI and the presence of VUR of the same level, or superior to IV (p=0.02), which was not observed in other levels of VUR. There was no association between the age at the date of the exam (<1 year old and ≥1 year old), gender, type of cystography and presence of antibiotic prophylaxis at the date of the exam.

**Table 4:** Association between demographic and clinical characteristics and occurrence of post-cystography urinary infection.

	With post-cystography UI	Without post-cystography UI^#^	p-value*
Age at the date of cystography	(n=23)	(n=508)	0.13
<1 year old	15 (65%)	250 (49%)	
≥1 year old	8 (35%)	258 (51%)	
Sex	(n=23)	(n=508)	0.44
Male	16 (70%)	313 (62%)	
Female	7 (30%)	195 (38%)	
Type of cystography	(n=23)	(n=508)	0.77
Radiological	11 (48%)	227 (45%)	
Isotopic	12 (52%)	281 (55%)	
Presence of VUR	(n=23)	(n=508)	0.05
Yes	14 (61%)	201 (40%)	
No	9 (39%)	307 (60%)	
VUR degree ≥IV	(n=14)	(n=201)	0.02
Yes	8 (57%)	57 (28%)	
No	6 (43%)	144 (72%)	
Antibiotic Prophylaxis	(n=23)	(n=508)	0.55
Yes	13 (57%)	255 (50%)	
No	10 (43%)	253 (50%)	

UI: urinary infection; VUR: vesicoureteral reflux; ^#^this is a presumptive data, since urine culture was only carried out with patients with symptomatology and routine urine analysis with suggestive urine; *Mann-Whitney U test of independent samples.

## DISCUSSION

After the bibliographic review, it appears that this is the first study in Portugal about the incidence of post-cystography UI, and the one with the largest sample, when compared to other international reviews.

The incidence of post-cystography UI found in this study was 4.3%, which is in agreement with the results of more recent studies, presenting values between 0 and 16%.^2^ ^,^ ^4^ ^,^ ^5^ ^,^ ^6^ ^,^ ^7^ ^,^ ^8^ Older articles referring to the 1970s present higher incidence rates, of up to 30%.^9^ ^,^ ^10^

In agreement with previous studies,^2^ ^,^ ^4^ there were more cases with evidence of VUR in the group that presented with UI (61%), when compared cases without UI (40%). Among the cases with VUR in the cystography, there was an association between post-cystography UI and the presence of VUR (level IV or higher) (p=0.02). This association was equally suggested by Visuri et al., even if not confirmed statistically.^5^

On the other hand, we did not find any association between the prescription of antibiotic chemoprophylaxis and post-cystography UI, which is in agreement with the most recent study about the topic, by Johnson et al., which showed that almost all patients with post-cystography UI were on antibiotic prophylaxis.^4^

In Hospital Garcia de Orta, bladder catheterization is conducted by different professionals, depending if it is a radiology or an isotope cystography. In the preparation for radiological cystography, a general pediatrics nurse performs bladder catheterization, whereas for isotope cystography, the procedure is carried out by nurses from the Nuclear Medicine Service, with experience in Pediatrics. This difference may lead to different incidences of post-cystography UI , but we did not observe any difference in our study. Johnson et al. reported similar results.^4^

As to the demographic characteristics of the analyzed population, similarly to this study, Johnson et al. also described the absence of association between sex or age at the exam (<1 year old and ≥1 year old) and the presence of post-cystography UI.^4^

This study has a few limitations. Because it is a retrospective study, it was not always possible to obtain detailed clinical data. Also, this study is a picture of one institution’s experience. When antibiotic prophylaxis was prescribed, its use could not be absolutely proven. It is important to mention that this study excluded patients with follow-up in other institutions, that only performed cystography in Hospital Garcia de Orta. The inclusion of these patients may have led to different results. Finally, there is always the possible bias that patients with UI symptomatology after undergoing a cystography did not come to our hospital, even though the parents were advised to look for the Pediatric Emergency or an appointment if they presented fever or post-cystography urinary symptoms.

Despite the described limitations, this study allowed to determine, for the first time in our country, the incidence of post-cystography UI in the pediatric age group. We considered that the development of prospective, multicenter, large-scale studies is essential to determine the risk factors for the occurrence of post-cystography UI, in order to guide the clinical practice and prevent this complication.

We reinforce the importance that bladder catheterization, necessary for this procedure, be carried out in the best conditions of asepsis. We also highlight the need for clinical surveillance of the patients after the exam, and of educating the parents and caretakers about the warning signs that should motivate the search for health services, especially in patients with evidence of VUR.

It is possible to conclude that, in this study, the incidence of post-cystography UI was relatively low (4.3%), and in agreement with more recent studies. There was an association between the occurrence of post-cystography UI and the presence of VUR. We highlight the importance of an adequate technique of bladder catheterization and the need for clinical surveillance after the exam. Further prospective and large-scale studies are necessary to establish the risk factors for the occurrence of post-cystography UI, and, therefore, to guide the clinical practice and prevent this complication.
